# Elevated perioptic lipocalin-type prostaglandin D synthase concentration in patients with idiopathic intracranial hypertension

**DOI:** 10.1093/braincomms/fcac240

**Published:** 2022-09-26

**Authors:** Achmed Pircher, Margherita Montali, Jatta Berberat, Andreas Huber, Neil R Miller, Thomas H Mader, C Robert Gibson, Albert Neutzner, Luca Remonda, Hanspeter E Killer

**Affiliations:** Department of Neuroscience/Ophthalmology, Uppsala University, Uppsala, Sweden; Department of Ophthalmology, San Bassiano Hospital, Bassano del Grappa, Italy; Department of Neuroradiology, Cantonal Hospital Aarau, Aarau, Switzerland; Department of Medicine, Private University in the Principality of Liechtenstein, Triesen, Liechtenstein; Wilmer Ophthalmological Institute, Johns Hopkins Hospital, Baltimore, MD, USA; COL(R) US Army, Moab, UT, USA; Coastal Eye Associates, Webster, TX, USA; KBR, Houston, TX, USA; Department of Biomedicine, University Hospital Basel & University Basel, Basel, Switzerland; Department of Neuroradiology, Cantonal Hospital Aarau, Aarau, Switzerland; Department of Biomedicine, University Hospital Basel & University Basel, Basel, Switzerland

**Keywords:** lipocalin-type prostaglandin D synthase, idiopathic intracranial hypertension, cerebrospinal fluid, optic nerve sheath compartment, papilloedema

## Abstract

The pathophysiology of vision loss and loss of visual field in patients with idiopathic intracranial hypertension with papilloedema is not fully understood. Although elevated CSF pressure induces damage to the optic nerve due to stasis of axoplasmic flow, there is no clear relationship between the severity of papilloedema and CSF pressure. Furthermore, there are cases of purely unilateral papilloedema and cases without papilloedema despite significantly elevated intracranial pressure as well as papilloedema that can persist despite a successfully lowered intracranial pressure. We hypothesize that at least in some of such cases, in addition to purely pressure-induced damage to the optic nerve, the biochemical composition of the CSF in the subarachnoid space surrounding the orbital optic nerve may play a role in the pathogenesis of vision loss. In this retrospective study, we report on lipocalin-type prostaglandin D synthase concentrations in the CSF within the perioptic and lumbar subarachnoid space in 14 patients with idiopathic intracranial hypertension (13 females, mean age 45 ± 13 years) with chronic persistent papilloedema resistant to maximum-tolerated medical therapy and visual impairment. CSF was collected from the subarachnoid space of the optic nerve during optic nerve sheath fenestration and from the lumbar subarachnoid space at the time of lumbar puncture. CSF was analysed for lipocalin-type prostaglandin D synthase and the concentrations compared between the two sites using nephelometry. The mean lipocalin-type prostaglandin D synthase concentration in the perioptic subarachnoid space was significantly higher compared with the concentration in the lumbar subarachnoid space (69 ± 51 mg/l without correction of serum contamination and 89 ± 67 mg/l after correction of serum contamination versus 23 ± 8 mg/l; *P* < 0.0001, Mann–Whitney U-test). These measurements demonstrate a change and imbalance in the biochemical environment of the optic nerve. Its possible effect is discussed.

## Teaser

Pircher *et al*. report significant higher concentrations of lipocalin-type prostaglandin D synthase in the perioptic subarachnoid space compared with the lumbar subarachnoid space. These findings indicate that next to cerebrospinal fluid pressure gradients, biochemical gradients might play a role in the pathophysiology of optic nerve damage in idiopathic intracranial hypertension.

## Graphical Abstract

**Graphical abstract:**
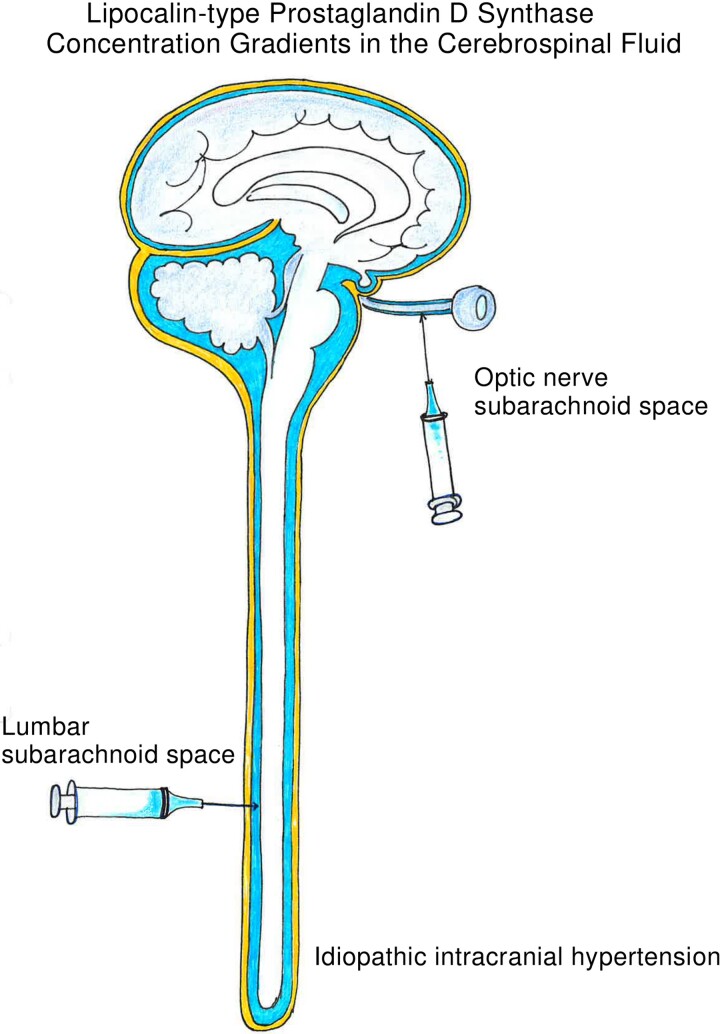

## Introduction

Idiopathic intracranial hypertension (IIH), also known as primary pseudotumor cerebri, is characterized by increased intracranial pressure (ICP) without any intracranial or spinal cord mass lesions and with normal-sized ventricles, normal CSF concentrations of protein and glucose, no cells in the CSF and, in most cases, papilloedema. This disorder affects predominantly women of childbearing age. In addition to headache, clinical symptoms are mainly visual disturbances [transient visual obscuration, visual field (VF) loss and binocular diplopia] and pulsatile tinnitus.^1^

Papilloedema is defined as swelling of the optic nerve (ON) head that results from stasis of axoplasmic flow leading to an accumulation of extracellular fluid due to elevated CSF pressure within the subarachnoid space (SAS) of the ON. The exact mechanism by which papilloedema causes visual disturbances is not fully understood. Although several studies have demonstrated CSF pressure–related damage to the ON due to stasis of axoplasmic flow, there is no clear relationship between the severity of papilloedema and CSF pressure.^2,3^

Papilloedema in patients with IIH is usually bilateral and symmetric, but some cases of bilateral markedly asymmetric papilloedema^4^ have been reported, such as a case of purely unilateral papilloedema verified by optical coherence tomography^5^ and even cases without papilloedema despite significantly elevated ICP.^6^ In addition, papilloedema sometimes persists despite a successfully lowered ICP.^7^ These phenomena suggest that ICP is not the only factor that plays a role in the pathophysiology of visual disturbances in the setting of papilloedema. The visual impairment that occurs in patients with IIH often is reversible if the papilloedema regresses but can become severe and irreversible, primarily if the papilloedema persists.^8^ It is not clear why optic disc oedema can persist despite normalized intracranial CSF pressure nor which mechanisms are causative for irreversible visual disturbances in patients with IIH and papilloedema.

A recently published study applying CT cisternography demonstrated disturbed CSF flow along the ON in patients with IIH who had persistent papilloedema.^9^ Specifically, CT cisternography in these patients demonstrated diminished contrast-loaded CSF within the SAS of the intraorbital ON with a minimum of contrast in the bulbar region of the ON just behind the lamina cribrosa. This finding is referred to as an ON sheath compartment syndrome. Due to compartmentation, the CSF pressure as well as the composition of CSF may differ from that in other CSF spaces within the compartmented ON sheath. The SAS compartmentation of the ON might not only disrupt the supply of newly produced CSF but also prevent the washout of potentially toxic substances. We performed an ON sheath fenestration (ONSF) in a series of patients with IIH and persistent papilloedema to allow outflow of accumulated CSF within the compartmented SAS in the retrobulbar ON. We also collected and examined the CSF released during this procedure for lipocalin-type prostaglandin D synthase (L-PGDS).

L-PGDS, also known as beta-trace protein, is a brain-specific glycoprotein and the most abundant brain-derived protein and second-most (after albumin) abundant protein in the CSF.^10^ It consists of 168 amino acids and belongs to the lipocalin superfamily. In the brain, L-PGDS mainly is expressed in the arachnoid membrane of the cells of the leptomeninges and the choroid plexus from which it is secreted into the CSF, although it also is expressed in oligodendrocytes and astrocytes.^11,12^ L-PGDS is a multifunctional protein involved in the regulation of various neurological processes. For example, altered L-PGDS expression levels in the brain are associated with various neurological disorders, including Alzheimer’s disease related and other types of dementia, multiple sclerosis, spinal canal stenosis and normal-pressure hydrocephalus^13-17^ and may play a role also in ocular diseases like open-angle glaucoma^18^ and normal-tension glaucoma.^19^ Recently, it has been demonstrated that L-PGDS is a major neuroprotective Aβ chaperone, suggesting that it plays a role in neurodegenerative processes.^20^ In non-neuronal tissues, the L-PGDS gene is expressed in the heart and in male genitalia and is present in body fluids like plasma, seminal fluid and urine.^21^ The concentration of L-PGDS is low (0.6 mg/l) in serum, and it therefore is used clinically for the diagnosis of suspected CSF leakage into the nasal cavity.^22^

In previous studies in patients with different ON disorders and in a non-homogeneous cohort of patients with papilloedema from various causes, we found an uneven distribution of L-PGDS between the CSF in the lumbar SAS and the CSF in the SAS surrounding the ON just posterior to the ocular globe.^23,24^ The current study compares the CSF concentration of L-PGDS between the lumbar and the perioptic SAS in a homogeneous cohort of patients with IIH and persistent papilloedema and visual impairment, in order to evaluate the biochemical milieu within the CSF surrounding the ON that might play a role in the pathophysiology of the optic neuropathy that can develop in patients with IIH and persistent papilloedema.

## Materials and methods

This retrospective study was approved by the local ethical commission (Ethikkommission Nordwest- und Zentralschweiz) and follows the tenets of the Declaration of Helsinki.

### Patients

From 2005 to 2020, 23 patients with the diagnosis of IIH were admitted to the department of ophthalmology because of therapy-resistant papilloedema and progressive visual impairment (visual acuity loss, VF defect or both) despite weight-loss attempts and maximum-tolerated therapy with systemic acetazolamide. Of these, 21 patients showed ON sheath compartmentation (reduced contrast agent in the SAS of the ON on cisternography), and 18 of 21 patients (3 patients refused) subsequently underwent ONSF. In 14 of the 18 patients, L-PGDS could be measured in both the ON and the lumbar SAS and were included in this study.

All patients initially underwent a full neuro-ophthalmological examination including best-corrected visual acuity, VF testing, testing of pupillary responses to light stimulation, slit-lamp biomicroscopy and ophthalmoscopic assessment. All patients also underwent MRI and lumbar puncture (LP) to establish the diagnosis of IIH. LP in all patients was performed in a standardized lateral decubitus position with a 20-gauge needle. The diagnosis was based on the updated modified Dandy criteria.^1^ Finally, all patients underwent CT cisternography (contrast agent allergies and renal insufficiency are the main contraindications) to assess CSF dynamics along the ON and to look for evidence of ON compartmentation. In cases with compartmentation, ONSF was offered and performed.

### Optic nerve sheath fenestration

CSF was sampled from the lumbar SAS during LP (L3–L4) and from the ON during ONSF. The LP was performed in a standard way in the lateral decubitus position using a 20-gauge spinal needle. The ONSF was performed via a medial transconjunctival orbitotomy under general anaesthesia. Special care was taken not to use fluids to moisten the cornea after the orbitotomy so as not to dilute the CSF. Multiple incisions were made in the dura of the ON with a 19-gauge blade, following which a piece of dura was excised. After the first incision, a gush of CSF was observed, followed by further slow CSF egress. CSF then was sampled with a syringe using a 37-gauge needle and immediately deep frozen.

### L-PGDS and albumin measurements

CSF from both sampling sites (lumbar and ON SAS) was examined for the content of L-PGDS and albumin. L-PGDS was chosen due to its abundance in the CSF and its exceedingly low serum concentration to avoid the possibility of false-positive results that often occur as a consequence of the presence of serum during CSF sampling. Albumin was measured to assess the amount of potential contamination of CSF with serum during the ONSF in the CSF samples from the ON SAS. Both L-PGDS and albumin levels also were measured in serum in order to estimate the amount of contamination with blood during ONSF. The concentration without blood contamination was calculated based on the albumin concentration measured in the lumbar SAS.^19^ Both concentrations, L-PGDS with (native) and without contamination in the perioptic SAS, are shown in Table 1. L-PGDS concentration measurements were performed by nephelometry on BNII (Dade Behring, Marburg, Germany) according to the manufacturer’s instructions. If necessary, samples were diluted so that the results would fall in the linear range. At least three measurements were performed on each sample, and the mean value was calculated.

**Table 1:** Demographics and clinical findings of IIH patients with papilloedema

IIH patients with papilloedema							
No.	Age m	Age f	Lumbar CSF-p (cmH_2_O)	Frisén scale	VF (dB)	Disease duration (months)	Symptoms
1		59	>25	1	9.2	17	BV, TVO, H
2		29	31	1	2.8	30	BV, TVO, H
3		56	34	2	0.9	45	BV, TVO
4		25	45	2	2.1	23	BV, TVO, H
5		48	37	4	2.9	17	BV, TVO, H
6		30	39	3	Goldmann	13	BV, TVO, H
7		61	34	1	12	16	BV, TVO, H
8		47	60	3	Goldmann	14	BV, TVO, H
9		43	>25	3	7.2	6	BV, TVO, H
10		40	>25	3	Goldmann	9	BV, TVO, H
11		20	43	2	Goldmann	11	BV, TVO, H
12		60	32	2	Goldmann	12	BV, TVO
13	57		47	2	Goldmann	5	BV, TVO, H, VI-P
14		48	31	2	Goldmann	14	BV, TVO, H
AM ± SD	57 ± 0	44 ± 13	39 ± 8		5.3 ± 3.8	17 ± 10	** **

Age for male (m) and female (f) in years. Lumbar cerebrospinal fluid pressure (CSF-p) (cmH_2_O) was measured by LP. Frisén scale at the time of LP/ONSF. VF MD measured/ONSF in months. Symptoms at the time of LP/ONSF in dB using standard automated perimetry. Disease duration defined as first diagnosis to time of LP. BV, blurred vision; TVO, transient visual obscuration; H, headache; VI-P, sixth nerve palsy; AM, arithmetic mean; SD, standard deviation.

### Statistical analysis

Statistical analysis was performed using SIGMAPLOT 10.0 (Systat Software, San Jose, CA, USA), Microsoft Excel 2010 (Microsoft Corporation, Redmond, WA, USA), and the SPSS 21.0 (IBM SPSS Inc., Chicago, IL, USA) for Windows statistical package. Data were analysed using the Mann–Whitney U-test because normal distribution was not given. All data are expressed as mean and standard deviation or median and interquartile ranges, respectively. Differences were considered significant when the error probability was *P* < 0.05.

### Data availability

The authors confirm that the data supporting the findings of this study are available within the article.

## Results

**Figure 1:**
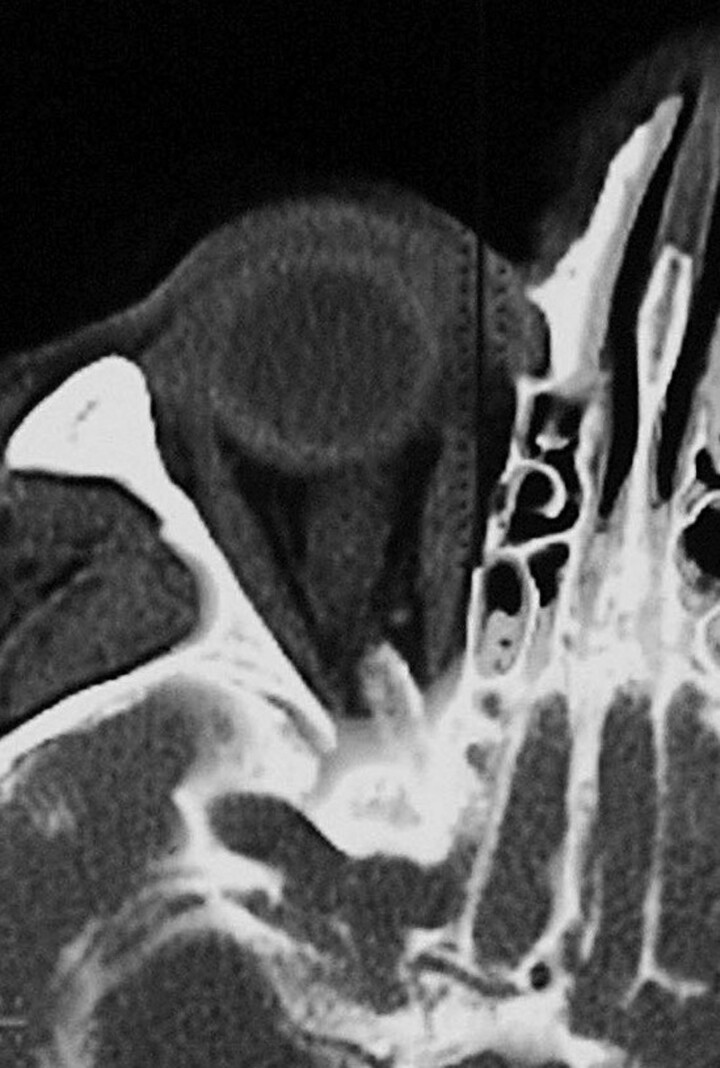
**Computer tomography assisted cisternography.** CT-assisted cisternography shows no flow of contrast medium into the intraorbital ON SAS in one of the included patients with idiopathic IIH and papilloedema.

Fourteen patients (mean age 45 ± 13 years), 13 female (44 ± 13 years) and 1 male (57 years), with IIH and persistent papilloedema underwent CT-assisted cisternography that demonstrated a markedly reduced concentration of contrast-loaded CSF compatible with ON compartmentation in one or both ONs (Fig. 1). These patients subsequently underwent unilateral or bilateral ONSF and the L-PGDS concentration in the CSF was measured in both the lumbar and perioptic SAS.

All 14 patients had visual symptoms, including blurred vision, transient visual obscurations, VF loss or a combination of these despite attempted weight loss and maximum-tolerated doses of acetazolamide. Twelve patients had headaches (more pronounced in recumbent position) and one patient reported diplopia that was due to a sixth nerve palsy. The time from diagnosis to CT cisternography varied from 5 to 45 months; the mean time was 17 ± 10 months. The time from CT cisternography to ONSF varied from 4 to 57 days; the mean time was 24 ± 17 days. At time of cisternography, all patients were being treated with oral acetazolamide (12 patients: 1500 mg/day, 1 patient: 1000 mg/day and 1 patient 500 mg/day due to side effects, mean dose: 1392 mg/day). MRI and CT demonstrated flattening of the posterior globe and distention of the ON sheath in all patients. In addition, three patients showed evidence of an empty sella. The mean body mass index was 33 ± 3. The mean visual acuity in the 14 patients using logMAR charts was 0.2. The mean VF mean deviation (MD) in seven of the patients using automated perimetry (SAP, Program G2 Octopus Haag-Streit, Switzerland) measured −5.3  dB; in the other seven patients, VFs were assessed using a Goldmann perimeter which showed enlarged blind spots, inferonasal VF cuts or general VF constrictions. The mean CSF opening pressure in 11 of the 14 patients measured by LP was 39 ± 8 cmH_2_O. In the remaining three patients, the lumbar CSF pressures were only documented to be >25 cmH_2_O and, therefore, were not included in the calculations. The clinical findings in the 14 patients in this study are detailed in Table 1.

ONSF was performed in 18 of 28 eyes in the cases in which cisternography demonstrated a distinct ONS compartmentation. Four patients underwent bilateral ONSFs, whereas 10 patients underwent unilateral ONSF. For the patients in whom bilateral ONSFs were performed, one randomly selected ON was used for statistical analysis. The randomize function in Microsoft Excel 2010 (Microsoft Corporation, Redmond, WA, USA) was used to randomly select the included side.

**Figure 2:**
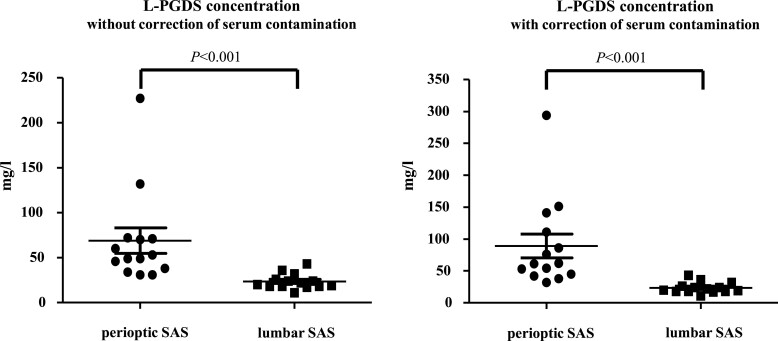
**Point plot of lipocalin-type prostaglandin D synthase concentrations.** Lipocalin-type prostaglandin D synthase concentrations in the CSF of the lumbar and perioptic SAS with and without serum contamination in idiopathic IIH patients with persistent papilloedema. Mann–Whitney U-test was used for data analysation.

The mean L-PGDS concentration measured 23 ± 8 mg/l (range, 11–43 mg/l) in the lumbar SAS, 69 ± 51 mg/l (range, 31–227 mg/l) in the perioptic SAS without correction of serum contamination and 89 ± 67 mg/l (range, 32–294 mg/l) in the perioptic SAS after correction of serum contamination (Table 2). There was a statistically significant difference in L-PGDS concentration between lumbar and perioptic SAS (*P* < 0.0001 without correction of serum contamination; *P* < 0.0001 with correction of serum contamination; Mann–Whitney U-test; Fig. 2). The mean albumin concentration measured 261 ± 166 mg/l in the lumbar SAS and 8152 ± 5400 mg/l in the perioptic SAS. The difference was statistically significant (*P* < 0.0001, Mann–Whitney U-test). The mean L-PGDS concentration in the serum measured 0.6 ± 0.1 mg/l. The mean albumin concentration in the serum measured 40 400 ± 2355 mg/l.

**Table 2:** L-PGDS concentrations in the lumbar and perioptic SAS with and without serum contamination in IIH patients with papilloedema

IIH patients with papilloedema					
No.	Age m	Age f	L-PGDS lumbar SAS (mg/l)	L-PGDS perioptic SAS (raw data with contamination) (mg/l)	L-PGDS perioptic SAS (without contamination) (mg/l)
1		59	22	53	54
2		29	26	31	38
3		56	32	71	141
4		25	19	38	45
5		48	20	46	62
6		30	43	49	53
7		61	18	70	76
8		47	17	60	86
9		43	11	34	42
10		40	24	227	294
11		20	18	49	61
12		60	36	31	32
13	57		24	72	111
14		48	18	132	151
AM ± SD	57 ± 0	44 ± 13	23 ± 8	69 ± 51	89 ± 67

L-PGDS was measured in the lumbar SAS at the level L3/L4 and in the perioptic SAS in the retrobulbar portion of the ON. Age for male (m) and female (f) in years. AM, arithmetic mean; SD, standard deviation.

## Discussion

The present study demonstrates a significantly higher concentration of L-PGDS in the CSF in the perioptic SAS compared with the concentration in the lumbar SAS and evidence of ON compartmentation in 14 IIH patients with persistent papilloedema. This finding indicates a biochemical alteration in the CSF surrounding the retrobulbar portion of the ON, with CSF flow along the ON being at least partially discontinuous from the intracranial CSF circulation. We consider that the significant difference in concentration of L-PGDS in the lumbar SAS and the SAS surrounding the ON results from disturbed CSF inflow and/or outflow, increased perioptic production of L-PGDS, or both. L-PGDS is a brain-derived protein that was chosen as a marker of altered CSF. Due to its abundance in the CSF, it might be representative for other brain-derived proteins in the CSF.

Well-regulated CSF flow is crucial for maintaining a continuous CSF exchange that allows the transport of nutrients to neurones, axons and glial cells as well as providing steady removal of potentially toxic waste products. Reduced perioptic CSF flow in the ONs of the patients in this study will therefore not only cause impaired inflow of newly produced CSF but also is likely to result in impaired removal of L-PGDS from the perioptic SAS with subsequent accumulation of L-PGDS as measured in the retrobulbar ON. This of course might not only apply to L-PGDS but also to other proteins, peptides, neurotransmitters and minerals in the CSF.

Papilloedema is a sequela of elevated ICP that is propagated via the optic canal into the perioptic SAS surrounding the orbital segment of the ON where it causes damage to the axoplasmic transport, leading to swelling of the ON axons and accumulation of extracellular fluid. However, in the 14 patients included in this series, the transmission of CSF pressure from intracranially to the eye is interrupted, which raises the question of what other mechanisms might be involved in optic disc swelling in these patients. We hypothesize that two mechanisms may be possible: (i) increased ICP that is partially transmitted along the ON despite the SAS compartmentation and (ii) increased mechanical pressure within the SAS of the ON due to compartmentation. These mechanisms also could explain the persistence of papilloedema despite lowered ICP due to weight loss and treatment with acetazolamide. Similar processes have been postulated to be responsible for the optic disc swelling and other ocular manifestations that occur in astronauts with ‘Spaceflight-Associated Neuro-ocular Syndrome’.^25^

Astronauts exposed to long-duration space flight have been documented to have optic disc oedema, globe flattening, choroidal folds and hyperopic shifts in refraction. This combination of findings has been explained as being due to a relative rise in ICP due to microgravity-related reduced venous outflow during long-duration space flight and compartmentation of CSF within the orbital SAS both during and after the flight.^26,27^ Indeed, three of the five astronauts in the original NASA 2011 study presented with optic disc swelling and ONS asymmetry suggestive of compartmentation.^26^ Cephalad fluid shifts and the fragile flow equilibrium in the densely septated cul-de-sac-like anatomic connection between the intracranial and orbital SAS, coupled with the pressure-induced growth of meningothelial cells within the lining the ONS, may lead to a one-way, ball-valve-like CSF flow and the gradual sequestration of CSF within the SAS surrounding the orbital portion of the ON in these astronauts similar to the patients in this study.^28^ This sequestration could result in locally elevated pressures within the ONS and the accumulation of toxic substances that could impact local structures with or without elevated brain CSF pressures.^28^ Asymmetric, chronic ONS compartmentation, possibly in conjunction with toxic metabolites, may have set the stage for tissue remodelling of the ON head and posterior sclera. Interestingly, persistent posterior globe flattening has been documented in several astronauts years following long-duration space flight, again suggesting that toxic chemicals may cause permanent sclera remodelling.^25^

The entire SAS of the ON is covered by meningothelial cells that form the arachnoid and pia layer as well as the septae and trabeculae that traverses the SAS. Meningothelial cells are sensitive to mechanical stimuli and react to pressure with proliferation.^29^ A possible scenario therefore could be that the elevated lumbar CSF pressure measured in patients with IIH and transmitted to the SAS of the ON leads to proliferation of meningothelial cells and, thus, to a narrowing and compartmentation of the ON SAS. Unlike the ICP, the exact pressure in such an orbital ON compartment is not known. Due to the small and complex anatomy of the SAS of the ON, reliable CSF pressure measurements in the orbital ON SAS are not yet possible; however, the enlarged ONS diameters in the retrobulbar ON seen on CT scans in our patients (Fig. 1) as well as in most patients with papilloedema from all causes suggests an increased local CSF pressure in their orbital ON SAS. As during ONSF after the first incision in the dural sheath, a gush of CSF invariably occurred, followed by a slow CSF egress, indicates increased pressure within the orbital ON SAS. Also, meningothelial cells were recently shown to take up amyloid beta, thereby likely acting neuroprotective.^30^ Altered CSF exchange in the ON might negatively impact meningothelial cell function in this area potentially also contributing to the observed visual impairment.

Protein toxicity is one of the main features in a variety of neurodegenerative diseases, such as Alzheimer’s disease, frontotemporal dementia, Parkinson’s disease, amyotrophic lateral sclerosis and Huntington’s disease.^31^ In these diseases, in addition to oligomerization and/or multimerization of associated toxic proteins, accumulation of disease-associated toxic proteins is a known pathological alteration.^32^ Accumulation of amyloid beta, for example, is thought to be a hallmark feature in Alzheimer’s disease. In all neurodegenerative diseases, cell death is the final outcome but is often preceded by clinical neurological deficits.^33,34^ L-PGDS, is a multifunctional protein that inhibits astrocyte proliferation and astrocyte mitochondrial ATP production.^35^ As astrocytes play an important role in the integrity of neurones, a high L-PGDS concentration could have a harmful effect on ON axons.

Next to toxic effects of L-PGDS, neuroprotective effects of L-PGDS are known as well. Recently, an *in vitro* study^20^ demonstrated a neuroprotective role of L-PGDS in Alzheimer’s disease. L-PGDS thereby acts as chaperone by inhibiting the primary and secondary nucleation of Aβ40. It interacts with monomers and the fibril surface and reduces thereby the final fibril content.^20^ These examples illustrate how complex the role of L-PGDS might be. L-PGDS concentrations in the lumbar CSF in the IIH patients in this study were in the same range as in other studies.^10,22^ The higher L-PGDS concentration in the perioptic SAS compared with those in the lumbar SAS in our patients may therefore also be a neuroprotective response to protect axons from potential damage from components of stagnant CSF and increased pressure and result therefore not only from disturbed CSF outflow but also from increased local (perioptic) production.

The albumin concentrations in both the lumbar SAS and the serum of the patients in this study are similar to those reported in several other studies.^36,37^ As the albumin concentration normally is much higher in serum than in CSF, the significant difference in albumin concentration between the lumbar SAS and the perioptic SAS in our patients is most likely due to contamination with blood during ONSF. The concentration of L-PGDS is, however, extremely low in serum. Thus, false-positive results caused by serum contamination are highly unlikely.

Clinical observations in patients with IIH suggest that in addition to purely pressure-induced damage to the ON, other pathophysiological mechanisms may be involved, among them ischaemic damage.^3^ Considering the large amount of CSF proteins that are recycled with a frequency of up to five times a day, we hypothesize that stagnant CSF with an accumulation of toxic components also might inflict damage to axons and glial cells of the ON, thus leading to visual disturbances. This hypothesis may be especially true for patients with persistent papilloedema, such as those in this study, as exposure time might play a crucial role.

Visual impairment in IIH patients often is reversible. The reversibility may be explained by the recovery of the obstructed axoplasmic flow when CSF pressure decreases. However, chronic papilloedema can also lead to irreversible VF constriction and, eventually, permanently reduced visual acuity. This is the case in pressure-induced ischaemia where the axons of the ON might be irreversible damaged, and recovery of visual symptoms is not possible. We hypothesize that a similar scenario may also occur as a consequence of neurotoxic substances that accumulate in the SAS surrounding the orbital portion of the ON. These substances could damage directly the integrity of ON axons, damage the vessels comprising the pial plexus of the ON leading to ON ischaemia or both. This, in turn, raises the question as to whether in IIH patients with persistent papilloedema, ONSF might be preferable to shunt procedures or stenting as it treats the problem more locally. All three procedures lower ICP, but only ONSF potentially could release stagnant CSF containing toxic substances from around the ON.

This study has several weaknesses. Firstly, there is no control group for obvious ethical reasons. However, a homogeneous distribution of particles (molecules and proteins) in a closed fluid system is the normal state according to the second law of thermodynamics. Secondly, the number of subjects in this study is small. Thirdly, the mean time difference between LP and ONSF was 24 ± 17 days, and we do not know if the CSF concentration of L-PGDS changed during this time. Fourthly, all patients had therapy-resistant papilloedema and severe ON-related visual disturbances and, thus, represent a specific population of IIH patients. Finally, we assessed only the concentration of L-PGDS in the lumbar and perioptic SAS. Ideally, the concentrations of other potentially toxic substances should be studied. Nevertheless, this study demonstrates a difference in the biochemical milieu between the lumbar SAS and the SAS of the ON in IIH patients with persistent papilloedema. Alteration in the biochemical content of CSF, in addition to pressure-induced ON damage, may play a role in the pathophysiology of the optic neuropathy that can occur in these patients and raises a question regarding the optimal treatment of such patients.

## Abbreviations

CT =: computed tomographic
ICP =: intracranial pressure
IIH =: idiopathic intracranial hypertension
L-PGDS =: lipocalin-type prostaglandin D synthase
MRI =: magnetic resonance imaging
ON =: optic nerve
ONSF =: optic nerve sheath fenestration
SAS =: subarachnoid space.
